# Analysis of spatial relationships in three dimensions: tools for the study of nerve cell patterning

**DOI:** 10.1186/1471-2202-9-68

**Published:** 2008-07-21

**Authors:** Stephen J Eglen, Dan D Lofgreen, Mary A Raven, Benjamin E Reese

**Affiliations:** 1 Cambridge Computational Biology Institute, Department of Applied Mathematics and Theoretical Physics, University of Cambridge, Cambridge, CB3 0WA, UK; 2 Neuroscience Research Institute and Department of Psychology, University of California at Santa Barbara, CA 93106-5060, USA

## Abstract

**Background:**

Multiple technologies have been brought to bear on understanding the three-dimensional morphology of individual neurons and glia within the brain, but little progress has been made on understanding the rules controlling cellular patterning. We describe new matlab-based software tools, now available to the scientific community, permitting the calculation of spatial statistics associated with 3D point patterns. The analyses are largely derived from the Delaunay tessellation of the field, including the nearest neighbor and Voronoi domain analyses, and from the spatial autocorrelogram.

**Results:**

Our tools enable the analysis of the spatial relationship between neurons within the central nervous system in 3D, and permit the modeling of these fields based on lattice-like simulations, and on simulations of minimal-distance spacing rules. Here we demonstrate the utility of our analysis methods to discriminate between two different simulated neuronal populations.

**Conclusion:**

Together, these tools can be used to reveal the presence of nerve cell patterning and to model its foundation, in turn informing on the potential developmental mechanisms that govern its establishment. Furthermore, in conjunction with analyses of dendritic morphology, they can be used to determine the degree of dendritic coverage within a volume of tissue exhibited by mature nerve cells.

## Background

Recent advances in developmental neuroscience have revealed how the mechanisms controlling proliferation, fate determination, migration, differentiation, synaptogenesis and cell death each contribute to the establishment of the architecture and connectivity of the mature brain. What has been lacking, however, is an understanding of the determinants of the positioning of neurons in 3D space: what controls the position a neuron will come to occupy within a brain structure relative to other cells in the local environment? In some structures, cells are packed side-by-side and there is no mystery to their spacing; in others, the distance between members of a cell type is often conspicuous, but we know nothing of the constraints imposing this spacing. We do not know, for instance, whether higher-order patterning is present, much like a rough or distorted lattice, or whether such cells are essentially randomly distributed within a volume of tissue, constrained only by the physical size of other cells. We may know quite a bit about the dendritic morphology of individual cells within a region, and can make inferences about the relationship between the morphology of a cell and how a population of those cells should be distributed to maximize a uniform dendritic sampling within the structure, but little concrete evidence exists to relate one to the other. This issue of neuronal positioning, almost completely neglected within the brain, has been explored extensively within the two-dimensional confines of the neural retina, where modeling studies have revealed the rules governing intercellular spacing, in turn suggesting plausible biological mechanisms that might embody such spacing rules [1-4]. Several mathematical techniques have been used to good effect in quantifying neuronal patterning in 2D [5-7]. Computer aided methodologies now allow for the localization of x, y, z centroids for neuroanatomical structures in 3D space [8,9], making the study of neuronal patterning in 3D space more feasible provided the tools for such spatial analysis exist.

To this end, we have now extended 2D methods to allow a comparable analysis on 3D datasets, described in this paper, to study the geometrical relationships between cells in a volume of tissue and to model their positioning in 3D space. To our knowledge, our program is the first GUI-driven program that allows for the interactive exploration and analysis of 3D neuronal datasets.

## Implementation

### Computing environment

Our program, *Spatial Analysis 3D (SA3D)*, is written mostly in matlab, for several reasons. First, matlab is cross-platform (available on Unix, Macintosh and Windows), and combines a powerful numerical environment with a portable GUI. We hope that some of our users will know the matlab language and hence develop further routines to be included in future releases of *SA3D*. Also, matlab can interface with programs written in other languages like C. We use this feature for some of the more numerically-intensive aspects of the computation (such as the D_min _model simulations), and also for reuse of existing C functions for the analysis of 3D datasets [10].

*SA3D *is available on our website [11] along with a separate user guide that describes in detail how to install and use the program.

### Data input/output

Data files (containing the x, y, z locations of neurons) can be read in to *SA3D *in several formats, most notably an excel data file, a comma separated value file, or a tab-delimited text file. Program output is mostly graphical, and standard matlab menu items allow for these windows to be printed. In addition, data generated by the program can be saved in the native matlab format – files are given the suffix .*sa3 *to indicate they are generated by *SA3D*.

### Analysis functions

The bulk of the program is dedicated to quantitative analysis of the spatial positioning of neurons, described below. In addition, we make use of matlab's built-in capacities for interactively viewing 3d datasets, e.g. to provide real-time rotation of datapoints [see Additional files 1 and 2].

#### Delaunay tessellation

Delaunay tessellations (and the complementary Voronoi domain volumes) are computed using the functions provided by matlab. These routines are, in turn, based upon the standard routines within Qhull [12], and can compute tessellations in geometrical spaces higher than just 2D.

#### Autocorrelation

The autocorrelation routines, and corresponding density recovery profile (DRP), are written in C, and generalise the formulation from 2D into 3D [5]. One complication with the calculation of the DRP in 3D is computation of the correction factor to handle boundary effects; here we have used the isotropic boundary correction [10], implemented in C by Prof. Adrian Baddeley along with the F, G, K functions.

#### F, G, K functions

The program also permits the plotting of three other statistics often used in the analysis of spatial point patterns, being the G, F and K functions [7,10]. The G function is the cumulative frequency distribution for the population of nearest neighbor distances:

$$G(t)=\frac{1}{n}\sum_{i=1}^{n}I(y_{i}\leq t)$$

where *y*_*i *_is the distance of cell *i *to its nearest neighbor, and *n *is the number of cells. *I*(*·*) is the indicator function, which is one if the argument is true, else zero. The F function, by contrast, plots the cumulative frequency distribution for the distance of each grid point to its nearest cell when a regularly spaced grid of *g *points is superimposed upon the population:

$$F(t)=\frac{1}{g}\sum_{i=1}^{g}I(z_{i}\leq t)$$

where *z*_*i *_is the distance of grid point *i *to the nearest cell. The F function is useful for detecting non-homogeneities in the distribution of cells within the field. Finally, the K function counts the expected number of cells within a given distance of a cell:

$$K(t)=\frac{\left|B\right|}{n^{2}}\sum_{i=1}^{n}\sum_{j\neq i}I(d_{ij}\leq t)$$

where |*B*| is the volume of the sample region and *d*_*ij *_is the distance between cell *i *and cell *j*. The K function is the cumulative version of the DRP. These three functions were implemented in C and kindly provided by Prof. Adrian Baddeley. All three functions include several methods to correct for edge effects; see [10] for details.

### Modeling functions

As well as analysing existing datasets, *SA3D *can generate synthetic datasets for comparison with experimental data. The program includes two main routines for generating sample points within a given volume:

• Random: neurons are positioned at random, subject to an optional constraint that no two neurons come closer than some minimal distance (denoted D_min_) to each other. The minimal distance is sampled from a Normal distribution with a given mean and standard deviation. Implementation of this rule follows that specified elsewhere [3] with the obvious extension into 3D. Neurons are added serially into the array until either the requested number *N *of neurons has been added or until the packing limit has been reached (in which case an error message is displayed).

• Hexagonal Close Packing (HCP): neurons are initially positioned into a regular hexagonal lattice, with a specified mean spacing between neurons; the number of neurons *N *is automatically determined so that the lattice fills the volume. The position of the neurons are then independently jittered by adding Gaussian noise with a specified standard deviation.

## Results and discussion

In this section, we present a case-study of usage of *SA3D*. We have used the program to generate two datasets, one using the D_min _model, and the other by jittering a regular hexagonal structure of points. Here we show how our different analysis techniques can reveal differences between the two datasets.

### Delaunay tessellation analysis

**Figure 1:**
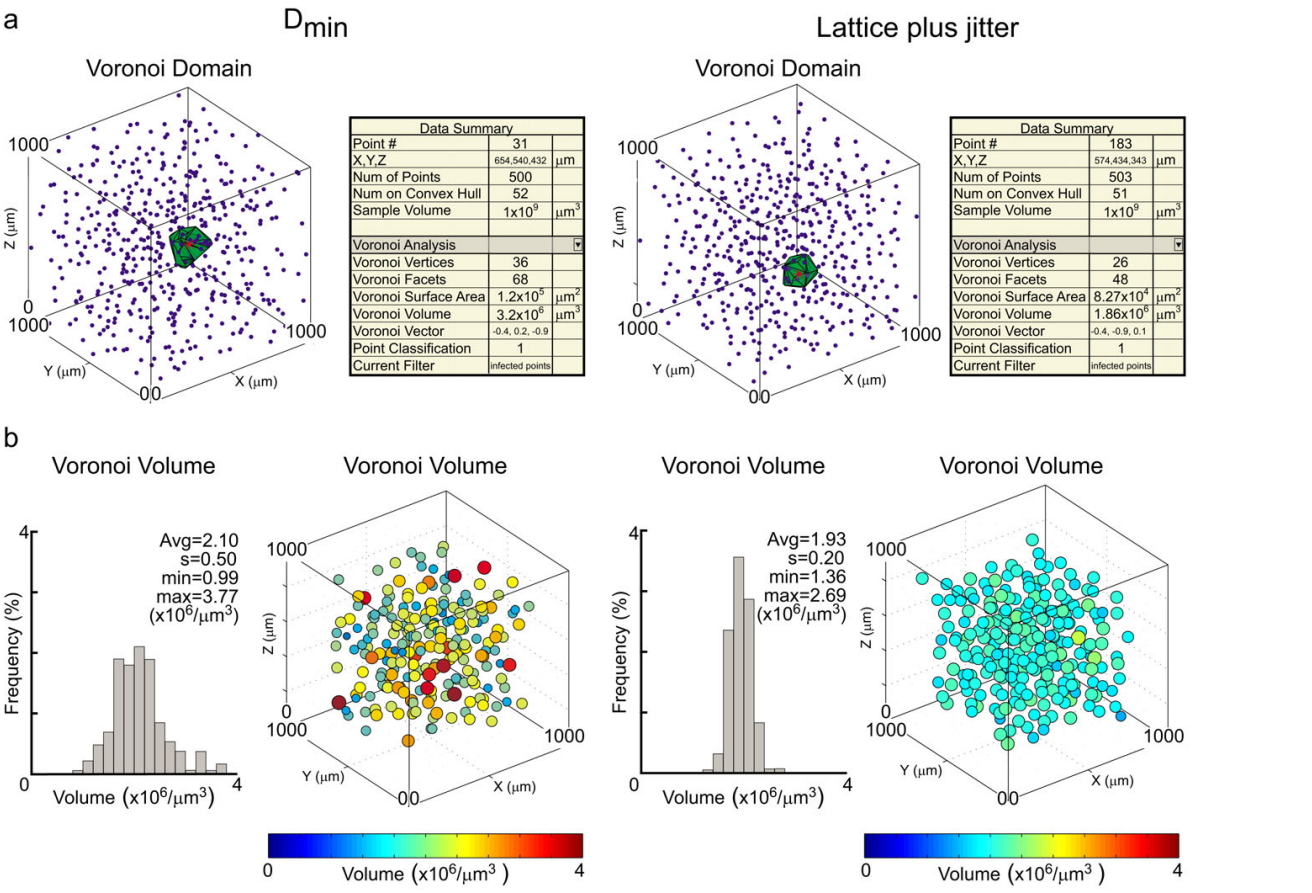
**Examples of two different simulated 3D neuronal populations and their Voronoi domain volumes**. a: 3D distributions of simulated populations of cells based on a minimal distance spacing rule (left) and on a jittered hexagonal lattice (right), with the Voronoi domain of a single cell illustrated within each field. The data summary panel to the right of each 3D distribution provides statistical details associated with the illustrated cell. b: Frequency distributions of Voronoi domain volumes associated with every cell (excluding those at the border) in the fields illustrated in a. The 3D depictions to the right of each frequency distribution portray the variation in Voronoi domain volume within space, where both color and increasing size signify an increase in volume. The histograms convey the population variability, while the bubble diagrams display that variability within 3D space.

By inputting x, y, z positional information for a population of cells, a variety of spatial statistics can be generated based upon the tessellation of the field by Delaunay tetrahedrons, including the derivation of Voronoi domains. For a sample of 500 points, these calculations are nearly instantaneous. Figure 1a shows examples of two simulated datasets of comparable density, one of randomly distributed cells constrained by a local minimal distance (D_min_) spacing rule, the other being a hexagonal lattice with local jitter applied to every cell. A single Voronoi domain of a given cell is illustrated for each case, along with its associated statistics to the right, including the volume of the Voronoi domain illustrated, the number of Voronoi vertices and facets for that domain, the sum of the surface area, and the vector details, being an index of its elongation within the field.

From this plot, one can select any individual point within the field for highlighting, or the points can be toggled sequentially by using the arrow keys. While the figure illustrates the Voronoi domain analysis, comparable analysis and presentation can be chosen for other derivatives of the Delaunay tessellation (e.g. the segment lengths, the tetrahedron volumes), including the nearest neighbor analysis.

For any of those analyses, the population statistics for every cell in the field can be plotted in one of two ways. Figure 1b illustrates these for the Voronoi analysis shown in figure 1a. The population of Voronoi domain volumes can be presented in histogram form, for all cells in the field, showing the less variable distribution for the jittered lattice relative to the D_min _simulation (figure 1b, left histograms in each pair). But to appreciate the nature of this variability across the field, a graphic representation of the Voronoi volume for each cell can also be plotted in 3D. All such 3D plots can be zoomed and rotated for detailed visualization [see Additional file 1].

Problematic boundary cells can be eliminated from the population statistics by selecting one of a number of increasingly stringent filters. Filter choice is dependent upon the analysis being conducted, since, for example, only those cells closer to the boundary than to any other cell will have an uncertain nearest neighbor, but cells meeting this restriction may still have an uncertain Delaunay tetrahedron or Voronoi domain.

### Spatial autocorrelation analysis

**Figure 2:**
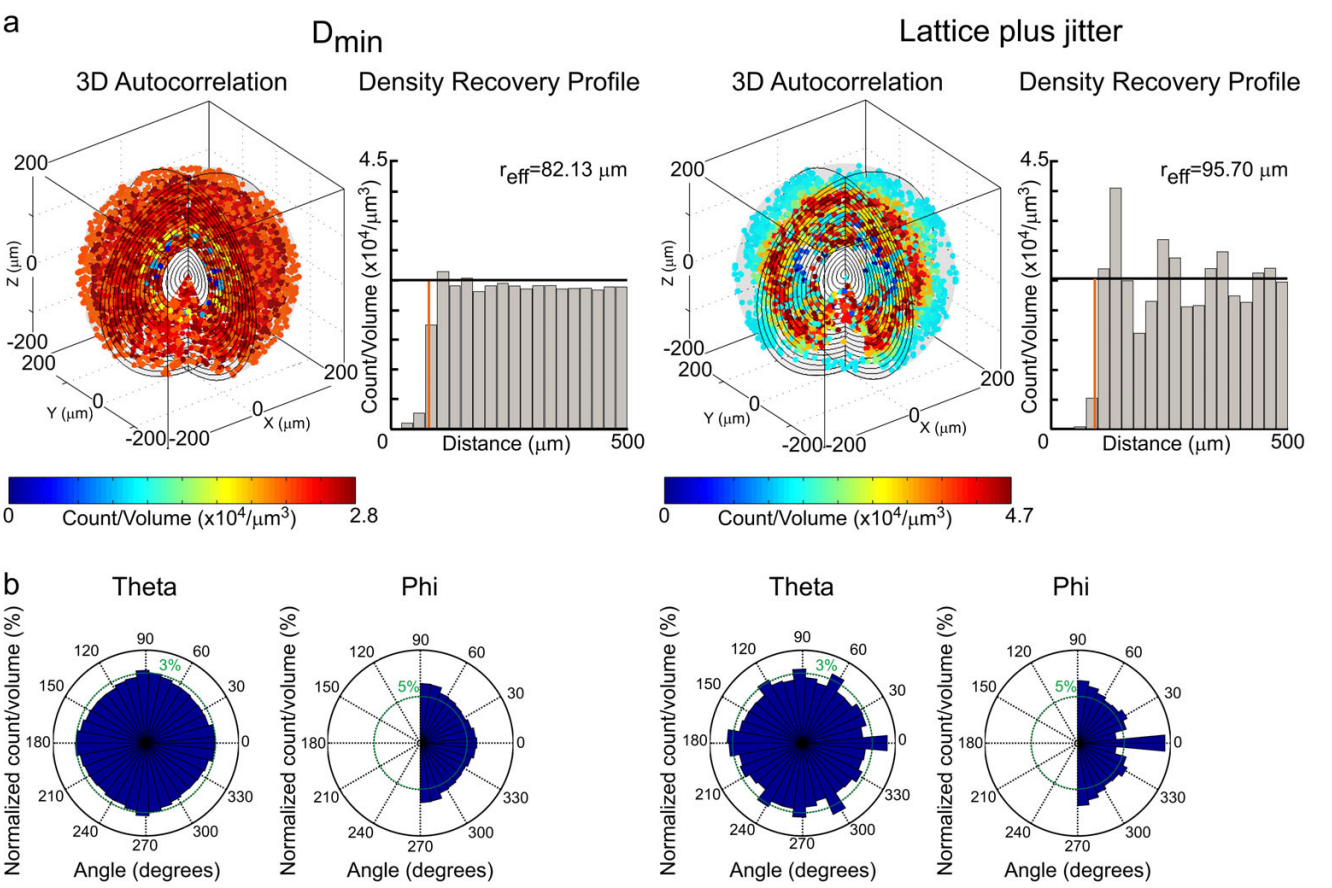
**Autocorrelation analysis of the two different simulated populations shown in Figure 1**. a: 3D spatial autocorrelograms for the fields shown in figure 1a. The minimal distance spacing rule (left) is revealed as a region surrounding the origin where cell density is reduced, displayed in histogram form to the right, in the DRP. The jittered lattice (right), in comparison, shows a periodic variation in cell density across the correlogram. The color of each point within the correlogram indicates the average density in the shell containing that point, indicated by the scalebar. Its DRP evidences the reduction in density surrounding the origin, but also reveals the waxing and waning in cell density as a function of increasing distance from the origin. The effective radius (r_eff_), the radius of the empty region surrounding the origin, is also indicated, by the red line. b: There is no angular variation in density for the minimal distance spacing rule, while these plots of azimuth and elevation for the jittered lattice display such variation evident within the correlogram.

The 3D spatial autocorrelation of the field can also be generated, from which the DRP is computed. The organizing principles of these two distributions of cells, while difficult to surmise by visual inspection of the population data or the tessellation analysis provided in figure 1, are now readily apparent: the minimal distance spacing rule is revealed by a region surrounding the origin of the autocorrelogram where cellular density is lower than at all other locations within the field; by contrast, the periodicity in the jittered hexagonal lattice reinforces itself within the correlogram, revealed as repeating regions of high and low cell density (figure 2a, left correlograms in each pair) [see Additional file 2]. The DRP can be computed from each correlogram [5], being a plot of average density in the correlogram as a function of increasing distance from the origin (figure 2a, right histograms in each pair), showing this difference clearly: the DRP for the D_min _simulation reveals only the presence of an exclusion zone surrounding the origin, whereas the DRP for the jittered lattice simulation reveals the waxing and waning of average density at increasing distances from the origin. As mentioned above, the program also permits the plotting of the K function, being the cumulative frequency histogram associated with the autocorrelogram.

The plots for both correlograms readily discriminate these features in histogram form (figure 2a); however, they conceal any angular variation in density. Those can be made apparent by separate plots of cell density as a function of their angles of azimuth and elevation (figure 2b): while the minimal distance spacing rule produces no systematic variation in density as a function of direction from the origin, the variation by angle is clearly revealed for the jittered lattice.

### Cumulative nearest neighbor distributions (G functions)

**Figure 3:**
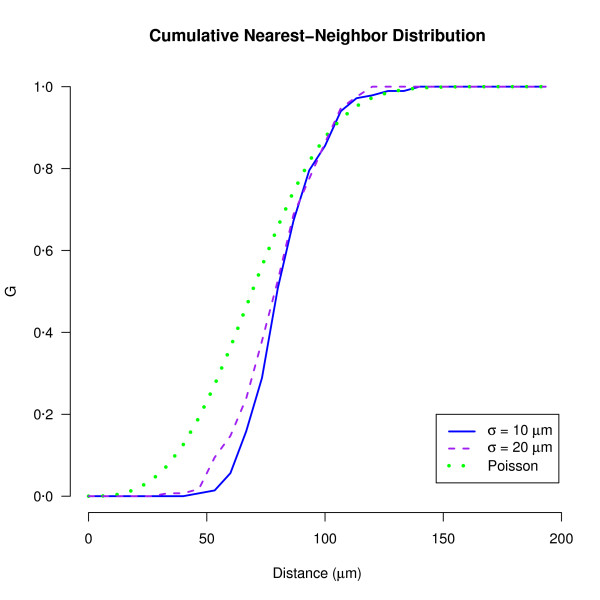
**G functions for D_min _simulations with slightly different exclusion zones**. Two simulated populations of 500 cells were created in a volume of 1000 × 1000 × 1000 *μ*m^3 ^using the D_min _model with a mean of 70 *μ*m and a standard deviation (*σ*) of either 10 *μ*m or 20 *μ*m. The G function for each simulated population is plotted, along with the expected G function if the cells were positioned according to complete spatial randomness (Poisson curve).

The F, G and K functions are commonly used to compare two sets of spatial patterns, or one set of patterns against the null hypothesis of complete spatial randomness (CSR). Here we demonstrate the utility of the G function to compare two simulated populations both created using the D_min _model, where only one parameter (the standard deviation of D_min _values, *σ*) differed (Figure 3). At small distances (*t <*80 *μ*m), *G*(*t*) for both simulated datasets is less than *G*(*t*) under CSR, which indicates exclusion in neuronal positioning. Further, *G*(*t*) helps discriminate between the two simulations: the sigmoidal curve for the D_min _simulation with *σ *= 20 *μ*m is wider than the curve for the simulation with *σ *= 10 *μ*m. This is expected, since the larger the standard deviation, the larger the range of minimal nearest-neighbor distances that are allowed in the model.

### Future directions

This paper accompanies the first release of *SA3D*, and we hope that it will continue to be developed over the coming years. Some features that we would like to implement in future releases include:

• Extension to multiple types of neuron. Currently the program assumes that all neurons are of the same type, and hence can be described just by their x, y, z location. The addition of an extra label, *t*, which typically would be an integer, would allow for us to discriminate between different types of neuron, and perform other analyses. For example, cross-correlation analysis could be used to test whether there is any spatial arrangement between neurons of different types. The corresponding 2D methods have previously been useful in helping assess whether groups of related retinal neurons should be regarded as one type or many types [13] and hence we expect these methods to be comparably useful in 3D.

• Model fitting. We currently provide two models for generating simulated neuronal populations; both these models require various parameters. Some automated method for finding the values of these parameters to minimize the discrepancy of the model and real data would be beneficial.

## Conclusion

These tools provide a means for investigating the rules governing nerve cell patterning within the central nervous system. They can be used to reveal the spatial statistics associated with a given population of cells; they can discriminate the patterning between experimental and control conditions; and they permit the modeling of real distributions based upon lattice-like distributions and on minimal distance spacing rules, both of which can be established with parameters modulated by the user. These spatial statistics can be compared with the morphological features associated with single cells, for example, by correlating the Voronoi domain with dendritic morphology in 3D, to understand the nature of the dendritic coverage within a volume of tissue [14]. A limitation of the analysis methods presented here is that they assume that each of the x, y, z dimensions are treated equally. Our methods are therefore inappropriate for specific cases, e.g. cortical microcolumns [15], where neurons of a certain type appear columnar across the depth of a slice; for these types of data, more specialised methods are appropriate [16]. However, even with columnar data, the interactive visualisation facilities offered by *SA3D *may still be useful for viewing the data. Studying the emerging regularity of a population of cells during development can clarify the potential biological mechanisms underlying it [17], in turn directing an assessment of the interactions that might mediate such homotypic cell spacing behavior. As well as informing us about developmental mechanisms, these methods can also be applied to adult tissue, as the spatial distribution of neurons can help assess the number of natural cell types in a structure [18]. The tools require the user to be able to assign positional information for each cell in 3D, and assume equal accuracy in the z dimension; this may be their greatest constraint, necessitating thick specimens sampled with confocal or two-photon microscopy, or sectioned specimens that can be accurately reassembled to preserve true spatial relationships in 3D. Ultimately, the benefit of such tools will depend upon the ingenuity of the researcher, but if the field of retinal research is anything to judge by, their adoption by brain researchers can only enlighten our understanding of the developmental neuroanatomy and nerve cell biology associated with such populations of cells.

## Availability and requirements

• Project name: Spatial Analysis 3D

• Project home page: 

• Operating system(s): Platform independent (tested on Windows XP, Linux, Mac OS X)

• Programming language: matlab/C

• Other requirements: matlab 7 or higher.

• License: *SA3D *(including the code by Prof. Baddeley) is distributed free under the conditions that (1) it shall not be incorporated in software that is subsequently sold; (2) the authorship of the software shall be acknowledged in any publication that uses results generated by the software; (3) this notice shall remain in place in each source file.

• Any restrictions to use by non-academics: none.

## Abbreviations

CSR: Complete Spatial Randomness; DRP: Density Recovery Profile; GUI: Graphical User Interface; HCP: Hexagonal Close Packing; *SA3D*: Spatial Analysis 3D

## Authors' contributions

SJE wrote the modeling code (D_min_/HCP), validated and tested the software, and refined both the manuscript and user guide. DDL designed, implemented and tested the program, including development of new analysis techniques and extension of existing methods into 3D, and wrote all program documentation. MAR specified the software requirements and tested the program. BER conceived the project, refined the software requirements, edited the user guide, and prepared the initial draft of the manuscript. All authors read and approved the final manuscript.

## Supplementary Material

Additional file 1 Rotations of example 3D populations. 3D plots of the populations of cells shown in figure 1a, in rotation, showing, for an individual cell near the center of each field, its near neighbors that define the Voronoi domain of that cell. The nearest neighbor is illustrated in red. Click here for file

Additional file 2 Rotations of example autocorrelograms. 3D autocorrelograms of the populations of cells shown in figure 2a, in rotation. Click here for file
